# Influence of Polishing and Glazing on Surface Characteristics and Biofilm Formation on Zirconia: An In Vitro Study

**DOI:** 10.3390/antibiotics14080739

**Published:** 2025-07-23

**Authors:** Gabriela de Arruda Ribeiro, Viviane de Cássia Oliveira, Adriana Cláudia Lápria Faria, Ana Paula Macedo, Carla Roberta de Oliveira Maciel, Cláudia Helena Lovato da Silva, Ricardo Faria Ribeiro, Renata Cristina Silveira Rodrigues

**Affiliations:** Department of Dental Materials and Prosthesis, Ribeirão Preto School of Dentistry, University of São Paulo, Café Avenue S/N, Ribeirão Preto 14040-904, SP, Brazil; gabrielaar@usp.br (G.d.A.R.); vivianecassia@usp.br (V.d.C.O.); adriclalf@forp.usp.br (A.C.L.F.); anapaula@forp.usp.br (A.P.M.); roberta.oliveira16@usp.br (C.R.d.O.M.); chl@forp.usp.br (C.H.L.d.S.); rribeiro@usp.br (R.F.R.)

**Keywords:** zirconia, biofilms, polishing, glazing, dental polishing, dental prosthesis, surface properties

## Abstract

**Background**: Monolithic zirconia has attracted considerable interest in dentistry due to its favorable physical and mechanical properties, making it a promising alternative for crown fabrication. Nonetheless, a standardized finishing protocol for this material has yet to be established. **Objective**: This study aimed to evaluate the surface characteristics and in vitro biofilm formation of zirconia finished by either polishing or glazing. **Methods**: A total of 72 zirconia specimens were fabricated and divided into control, glazing, and polishing groups. Surface analysis included roughness, wettability, and surface free energy. Microbiological analysis included CFU (colony-forming units per mL) counts, microbial adhesion at 2, 4, 6, and 8 h, biofilm biovolume, and qualitative biofilm assessment via scanning electron microscopy (sEm). **Results**: The glazing group showed significantly greater roughness than the polishing (*p* = 0.006) and control (*p* = 0.016) groups, along with a lower contact angle (polishing—*p* = 0.002; control—*p* < 0.001) and higher surface energy (polishing—*p* = 0.005; control—*p* < 0.001). No significant differences were observed in CFU counts for the tested microorganisms (*C. albicans*, *p* = 0.158; *L. casei*, *p* = 0.610; *S. mutans*, *p* = 0.904). Regarding microbial adhesion, the polishing group showed a smaller biofilm-covered area compared to the control group for both total biofilm (*p* = 0.008) and viable biofilm (*p* = 0.005). no statistically significant difference was observed in biofilm biovolume (*p* = 0.082). **Conclusions**: These findings suggest that, despite the surface differences among the groups, biofilm formation was not significantly affected.

## 1. Introduction

The use of zirconia on prosthetic dental restorations has gained considerable importance due to its excellent mechanical and esthetic properties [1]. However, to ensure the long-term success of these restorations, it is essential to comprehend the surface characteristics of the material, such as roughness, wettability, and surface energy, as these factors can directly affect bacterial adhesion and biofilm formation. Rough surfaces, for instance, may facilitate bacterial adhesion and biofilm formation, potentially leading to alterations in optical properties and complications including gingival inflammation and dental caries, in addition to contributing to the wear of the opposite dentition [2,3,4,5,6,7].

Wettability and surface energy also play a crucial role in this context. Surfaces exhibiting moderate wettability facilitate bacterial adhesion [8], and the surface free energy influences the adsorption of biomolecules and the bacterial adhesion [8]. These factors make the finishing of restorations a key factor in minimizing biofilm accumulation, enhancing esthetics, and promoting oral health [9].

Polishing and glazing are the primary methods employed for zirconia surface finishing. Whereas glazing is performed by applying a thin layer of a vitreous material to zirconia, resulting in improved esthetic properties, polishing is carried out using a sequential series of abrasive burs of decreasing grit sizes, providing surface smoothness and acting as an alternative approach to surface-level finishing zirconia [6,10]. However, a standardized method for this material finish and polish still does not exist [1]. Moreover, there is limited knowledge about which method favors lower microbial adhesion, with divergences among the results reported in recent studies [11,12,13].

Given the clinical importance of biofilm formation and bacterial adhesion, it is essential to investigate the relationship between zirconia superficial finishes and bacterial colonization. Microbial adhesion can compromise a restoration’s longevity, causing failures such as secondary caries in natural teeth or peri-implantitis in dental implants [14]. Therefore, it is crucial to understand which surface treatment yields better outcomes, ensuring greater durability, esthetics and oral health for patients [9]. Thus, the objective of this study was to analyze the surface characteristics and biofilm formation on zirconia subjected to polishing or glazing.

## 2. Results

### 2.1. Surface Analyses

**Figure 1:**
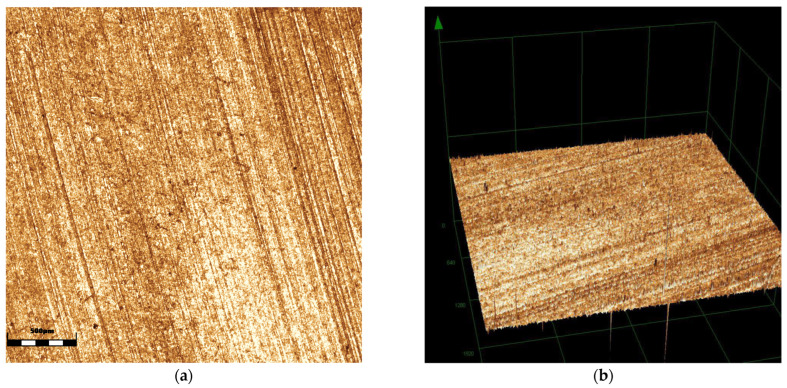
Control group: (**a**) 2D image; (**b**) 3D reconstruction image.

**Figure 2:**
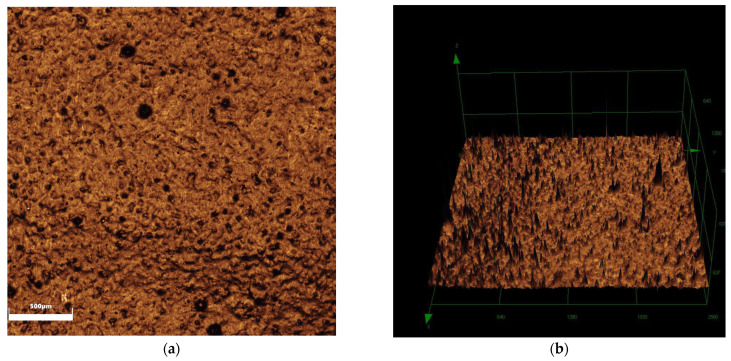
Glazing group: (**a**) 2D image; (**b**) 3D reconstruction image.

**Figure 3:**
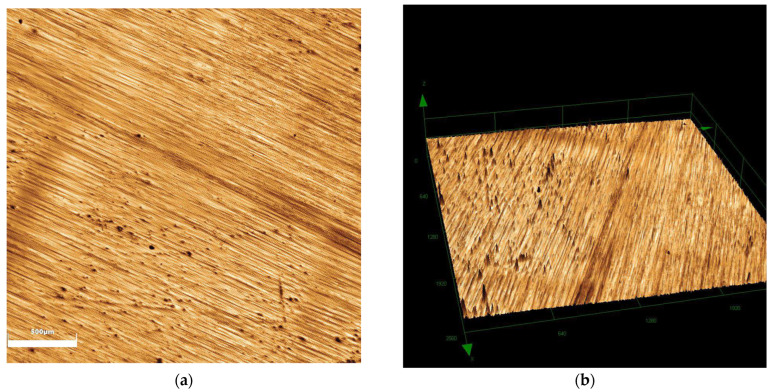
Polishing group: (**a**) 2D image; (**b**) 3D reconstruction image.

When comparing surface roughness, a significant difference was observed among the groups (*p* < 0.001), with the glazing group exhibiting greater roughness compared to the polishing (*p* < 0.006) and control groups (*p* = 0.016), as shown in Table 1. In terms of wettability, the glazing group exhibited lower values (polishing—*p* = 0.002; control—*p* < 0.001). Figure 1, Figure 2 and Figure 3 show the images obtained for surface roughness using confocal laser scanning microscopy.

The results obtained for surface energy and its polar and dispersive components are presented in Table 2. A statistically significant difference was observed for surface energy (*p* = 0.009), dispersive energy (*p* < 0.001), and polar energy (*p* < 0.001).

### 2.2. Microbial Load (CFU) and Biovolume

No significant difference was found for any of the tested microorganisms (*C. albicans*—*p* = 0.158; *L. casei*—*p* = 0.610 and *S. mutans*—*p* = 0.904). Statistical analysis of biovolume also revealed no significant differences (*p* = 0.082).

### 2.3. Microbial Adhesion

Table 3 presents the descriptive statistics of microbial adhesion assays at 2, 4, 6 and 8 h for the total microorganisms while Table 4 shows the data for viable microorganisms and Table 5 shows the data for dead microorganisms.

For microbial adhesion, time (*p* = 0.002), Group (*p* = 0.001), condition (viable or total microorganisms) (*p* < 0.001) and the group × condition interaction (*p* = 0.008) were statistically significant. Other observed interactions were not significant. Regarding time, a duration of 2 h showed a lower amount of biofilm compared to durations of 4 h (*p* = 0.008), 6 h (*p* = 0.038) and 8 h (*p* = 0.014). No significant differences were found between 4, 6 and 8 h. Evaluating the group × condition interaction when comparing condition with the same group, the polishing group showed a lower biofilm-covered area than the control group for both total biofilm (*p* = 0.008) and viable biofilm (*p* = 0.005). Comparing conditions within each group, the total biofilm was greater than the viable biofilm for all groups (polishing—*p* = 0.003; glaze—*p* < 0.001; and control—*p* = 0.001). The microbial adherence of dead biofilm showed a statistically significant interaction between group and time (*p* = 0.026). At 4 h, the polishing group exhibited a significantly higher amount of adhered dead biofilm compared to the control (*p* = 0.0017) and glazing groups (*p* = 0.007).

### 2.4. Scanning Electron Microscopy

**Figure 4:**
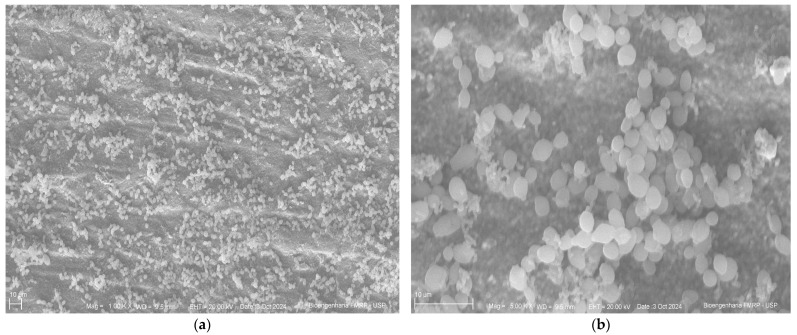
Control group (**a**) at 1000× magnification; (**b**) at 500×.

**Figure 5:**
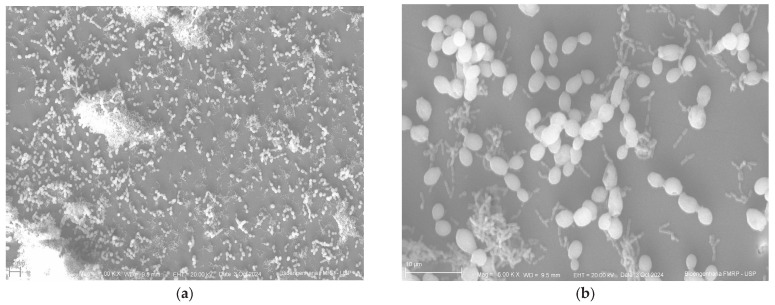
Glazing group (**a**) at 1000× magnification; (**b**) at 500×.

**Figure 6:**
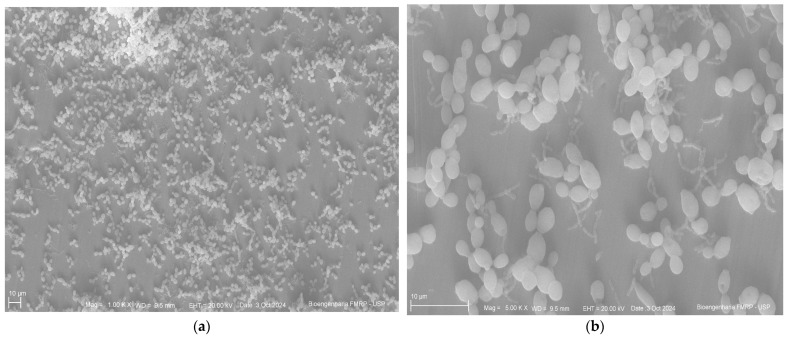
Polishing group (**a**) at 1000× magnification; (**b**) at 500×.

Below are the images obtained through scanning electron microscopy from the glazing, control and polishing groups (Figure 4, Figure 5 and Figure 6).

## 3. Discussion

Considering the importance of surface characteristics in bacterial adhesion, this study analyzed the effects of polishing and glazing on surface roughness and biofilm formation on monolithic zirconia. Based on the results obtained, it was found that the tested surface finishing methods influenced surface properties—roughness, wettability, and surface free energy. However, no statistically significant differences were observed regarding biofilm formation on this material.

According to the results, the glazing group exhibited higher roughness values compared to the control and polishing groups. Previous studies have also reported that zirconia subjected to glazing presents higher surface roughness compared to zirconia subjected to polishing, in agreement with the findings of the present study [11,15,16,17,18]. Similarly, [19] also observed greater roughness in the glazed group, reinforcing the trend that this procedure increases surface irregularities. Other studies suggest that polishing may be an effective alternative for achieving smoother surfaces, thereby reducing biofilm accumulation [6,20]. However, although higher surface roughness is often associated with increased microbial adhesion, our data did not demonstrate a significant impact on biofilm formation. This suggests that roughness alone may not be the primary determinant of microbial adhesion, especially under in vitro conditions.

Surface roughness values above 0.2 μm are considered more conducive to microbial colonization and biofilm formation, factors that can lead to gingival inflammation, secondary caries, and restoration failure [2,9]. In our study, the glazing group showed significantly higher roughness than the polishing and control groups; however, all groups exceeded this threshold. Nevertheless, these differences in roughness did not translate into a significant increase in biofilm formation in vitro.

A material’s hydrophilic or hydrophobic nature has a direct and significant influence on microbial adhesion to its surface. Based on the results from the wettability and surface free energy analyses, although the glazing group showed statistically significant differences compared to the polishing and control groups, all groups exhibited hydrophilic behavior. In line with the present study, Dal Piva et al. (2018) [11] and Alves et al. (2019) [16] also reported hydrophilic behavior for both surfaces when evaluating surface free energy and wettability of zirconia specimens subjected to polishing and glazing. This characteristic—defined by the tendency to attract water molecules—may have relevant implications, such as promoting microbial adhesion to the material’s surface. Moreover, as noted by Alves et al. (2019) [16], higher wettability values may be associated with reduced antagonist wear by improving lubrication during contact, thereby decreasing wear between contacting surfaces.

The microorganisms selected for in vitro biofilm formation in this study (*S. mutans*, *C. albicans*, and *L. casei*) are closely associated with dental caries and are commonly found in the oral cavity [21,22,23,24,25,26,27,28,29,30]. *S. mutans* is considered the main etiological agent of caries, while *C. albicans* is the most prevalent fungus in the oral cavity and interacts synergistically with *S. mutans*, enhancing the cariogenicity of the dental biofilm [24,25,26]. Additionally, *Streptococcus* and *Lactobacillus* act as primary colonizers, promoting the adhesion of other microorganisms to the acquired pellicle, and *L. casei* exhibits greater adhesion capacity when associated with *S. mutans* [31,32].

Few studies have compared biofilm formation and microbial adhesion on different zirconia surface finishes. Dal Piva et al. (2018) [11] evaluated microbial load on zirconia specimens subjected to polishing or glazing by counting colony-forming units. Contrary to our findings, which showed no statistically significant differences, they reported greater colony growth on glazed surfaces compared to polished ones. They also found that surface roughness was a determining factor in biofilm formation, with glazed surfaces exhibiting higher roughness values than polished ones. These findings contrast our results, as we did not observe increased biofilm formation in the glazing group. Among the microorganisms tested in the multispecies biofilm (*S. sanguinis*, *S. mutans* and *C. albicans*), higher growth of *S. mutans* was observed regardless of surface type. This may be explained by the role of *S. sanguinis* as a facilitator for the growth of other *Streptococcus* species, and that of *C. albicans* as a promoter of *S. mutans* adhesion [11].

Cepic et al. (2020) [19] examined microbial adhesion by quantifying colony-forming units of *C. albicans* in biofilms formed on monolithic zirconia surfaces subjected to polishing and glazing protocols. Similarly to the present study, no statistically significant differences were observed between the groups regarding *C. albicans* adhesion. However, they found that pre-wetting the samples with saliva reduced *C. albicans* adhesion compared to protocols without pre-wetting. This finding aligns with those of Koch et al. (2013) [33], who previously reported that saliva has a homogenizing effect on the surface properties of materials with different surface free energies, and with those of Okada et al. (2008) [34], who demonstrated that surface hydrophilization inhibits biofilm formation. Like the study by Cepic et al. (2020) [19], this study also used artificial saliva to moisten the specimens, simulating the acquired pellicle. However, further studies on protein adsorption, formation, and the role of the acquired pellicle are encouraged.

Poole et al. (2020) [35] investigated the adhesion of *Prevotella intermedia* to different ceramics (leucite-based glass–ceramic, lithium disilicate-based glass–ceramic, zirconia-reinforced lithium silicate glass–ceramic, and monolithic zirconia) after polishing and glazing. Their results showed no statistically significant differences in CFU counts; however, a significant increase in surface roughness was observed in the glazed group compared to the polished one. These findings are consistent with those of the present study.

Scanning electron microscopy images revealed a similar pattern of microbial adhesion across the surfaces of all tested groups, supporting the quantitative analysis results. Additionally, the images showed greater surface irregularities in the control group and a more homogeneous surface in the polished group, further supporting the findings. Despite these visual observations, the microbiological results were not statistically significant, limiting their clinical generalizability.

Despite the relevant findings, this study presents some limitations that should be considered when interpreting the results. The analysis of biofilm formation was conducted under in vitro conditions, which do not fully replicate the complex and dynamic environment of the oral cavity, including variations in salivary flow and composition, pH, temperature, masticatory forces, individual diet, and oral hygiene practices—factors that can significantly influence microbial adhesion and biofilm maturation. In particular, key clinical variables such as shear stress from salivary flow or mastication, and dietary components, which can modulate microbial behavior and biofilm development, were not accounted for and may alter the outcomes observed under laboratory conditions. Moreover, this study was limited to a single incubation period and a restricted number of microorganisms; therefore, the conditions did not reflect the microbial diversity typically found in the oral environment. Therefore, although the results obtained are relevant, caution is recommended when extrapolating them to intraoral conditions. Future studies using in situ or in vivo models are important to complement the current findings and to more comprehensively assess their clinical applicability. Additionally, surface chemical analyses (such as XPS or FTIR), protein adsorption and acquired pellicle formation assessments, EPS characterization, CLSM with time lapses and studies evaluating the effects of thermal or mechanical aging on zirconia surfaces should be conducted to broaden the scope of the discussion.

Nevertheless, no statistically significant differences in biofilm formation were observed among the tested groups, suggesting that additional factors also play a determining role in this process. Polishing proved effective in producing smoother surfaces, which may offer advantages in terms of mechanical performance and reduced antagonist wear. From a microbiological standpoint, however, it is not possible to assert—based on the present data—that polishing offers clinical advantages in terms of reduced bacterial colonization.

## 4. Materials and Methods

**Figure 7:**
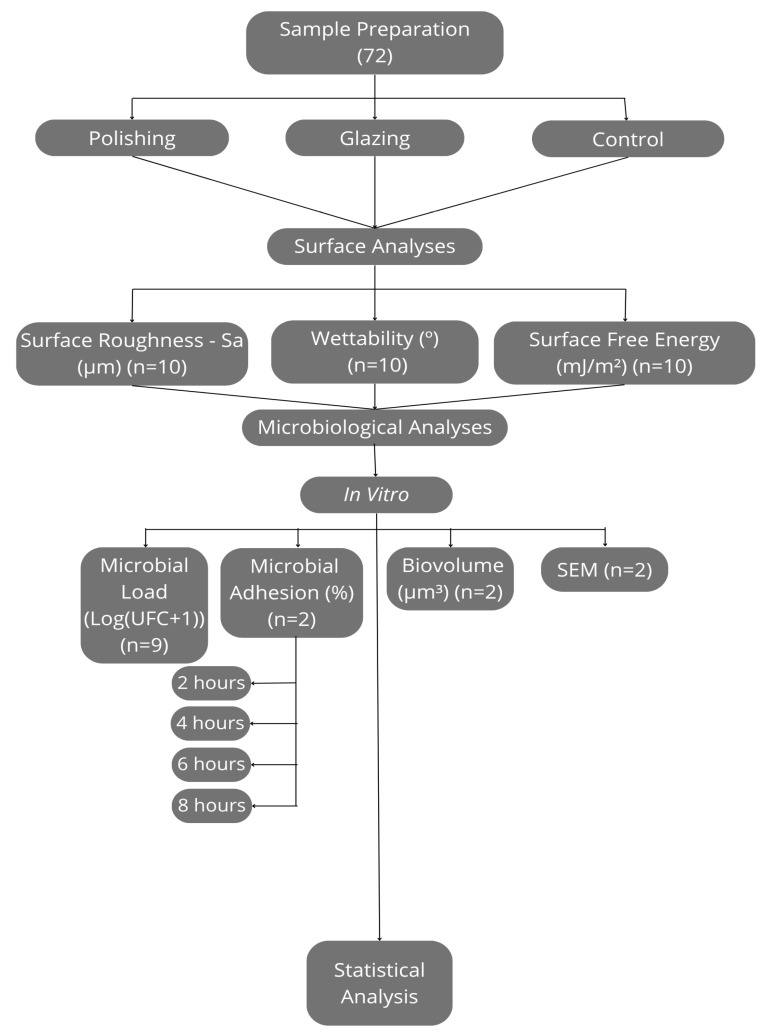
Experimental protocol flowchart.

The sample size for surface analyses was determined based on the data from Cepic et al. (2020) [19] using OpenEpi-Toolkit Shell for Developing New Applications (available online: https://www.openepi.com/SampleSize/SSMean.htm, accessed on 29 May 2025) with *n* = 10 being recommended. For microbiological analyses, *n* = 9 was used [36,37,38,39], performed in biological triplicate, as recommended in the literature for colony-forming unit calculations. Additionally, *n* = 2 was used for scanning electron microscopy (SEM), biovolume [40] and the microbial adherence of viable and dead microorganisms after 2, 4, 6 and 8 h [41]. The experimental protocol is summarized in Figure 7.

### 4.1. Specimen Preparation

In total, 72 monolithic zirconia specimens (Ceramill Zolid FX Preshade, Amann Girrbach, Koblach, Austria) were fabricated in square shapes (10 mm × 10 mm × 2 mm). The zirconia block was sectioned using a precision saw (Isomet 1000 Precision Saw, Buehler, IL, USA) and manually finished via sequential wet sanding using silicon carbide papers of grift sizes 400, 600, 800 and 1200 to achieve the predetermined dimensions, accounting for volumetric shrinkage after sintering and to ensure surface smoothness. Dimensions were verified with a digital caliper (Mitutoyo, Kanagawa, Japan). Specimens were sintered in a furnace (inFire HTC Speed, Sirona, Bensheim, Germany) following the manufacturer’s instructions.

Surface finishing was performed by a single operator. Polishing was carried out using a medium-grift green diamond polisher (Diacera W16DCmf, Eve Ernst Vetter GmbH, Birkenfeld, Germany) for 1 min, followed by a fine-grift pink diamond polisher for high gloss (Diacera W16DC, Eve Ernst Vetter GmbH, Germany) for 30 s.

For this procedure, the specimens were positioned in a metal matrix to ensure stabilization, and the polishers were attached to a handpiece connected to a micromotor (SL30, Dabi Atlante, Ribeirão Preto, Brazil) operating at a low speed, ranging from 7000 to 12,000 rpm (rotation per minute), under dry conditions. A modified paralleling device was employed to position both the material matrix and the rotary instrument, ensuring proper parallelism and standardization of the load applied to all specimens [42]. This device allows for precise control of the distance between the polisher and the specimen through a millimeter scale, as well as horizontal movement via a manual crank [43].

The glaze (Glaze InSync, InSync, San Jose, CA, USA) was applied in a single layer using a brush, following the manufacturer’s instructions. Firing was carried out in a furnace (Sinter Press Alumini, EDG, Sao Carlos, Brazil), also in accordance with the manufacturer’s instructions.

The control group did not undergo any surface finishing.

### 4.2. Surface Roughness (Sa)

Images of the zirconia specimens (*n* = 10) were captured using a laser scanning confocal microscope (LEXT OLS4000, Olympus, Westborough, MA, USA) at 5× magnification. Surface roughness (Sa) was quantified through software (LEXT 3D Measuring Laser Microscope OLS4000, Olympus, Tokyo, Japan).

### 4.3. Wettability and Surface Free Energy

The wettability of the samples (*n* = 10) was assessed by the sessile drop method, measuring the contact angles (θ) (°) of distilled water on the zirconia specimen surface using a goniometer (SCA20-DataPhysics Instruments GmbH, Filderstadt, Baden-Württemberg, Germany). For this, a 5 µL droplet was placed on the specimen surface, and an image was captured by a charge-coupled device (CCD) camera for contact angle calculation through a software (OCA-20, OneAttension, Biolin Scientific Inc., Manchester, Northwest, UK). Wettability was determined by calculating the arithmetic mean of the contact angles obtained from three droplets applied to each specimen. Between measurements, specimens were dried with compressed air [38].

Surface free energy (*γ_s_*) (mJ/m^2^) (*n* = 10) was determined by measuring the contact angles of the three liquids (5 µL each)—diiodomethane, distilled water, and formamide—on the zirconia specimen surfaces. Surface free energy was calculated using the Owens–Wendt–Kaeble equation [44] via OCA-20 software (OneAttension, Biolin Scientific Inc., Manchester, Northwest, UK).

### 4.4. In Vitro Microbiological Analysis

Specimens were sterilized by exposing each side to UV radiation for 20 min [45]. The ATCC (American Type Culture Collection) strains of the microorganisms *Candida albicans* (ATCC 10231), *Streptococcus mutans* (ATCC 25175), and *Lactobacillus casei* (ATCC 334) were used in the assays. Each strain was thawed and cultured in its respective specific growth medium [*C. albicans*: Sabouraud Dextrose Agar (SDA); *S. mutans* and *L. casei*: Brain Heart Infusion Agar (BHI)] for 48 h. Subsequently, a colony was transferred to the broth and incubated for 24 h at 37 °C. The culture was then centrifuged at 4200× *g* for 5 min and washed twice with 10 mL of Phosphate-Buffered Saline (PBS). The inoculum concentration was standardized to 10^8^ CFU/mL for *L. casei* and *S. mutans*, and 10^6^ CFU/mL for *C. albicans*.

Specimens were distributed into 24-well culture plates. To simulate the acquired pellicle formation, 1.5 mL of artificial saliva (composition: NaCl (400 mg/L), KCl (400 mg/L), CaCl_2_·H_2_O (795 mg/L), Modified Fusaya-Artificial Sa-NaH_2_PO_4_·H_2_O (690 mg/L), NaS·9H_2_O (5 mg/L), urea (1000 ma’s liva mg/L), and artificial saliva (AS) (Sigma Chemical Company, St. Louis, MO, USA) was added to each well, and the plates were incubated at 37 °C with agitation at 75 rpm for 2 h [46]. For microbial adhesion analyses, each well was filled with 1.5 mL of the inoculated culture medium, and the plates were incubated again under the same conditions. Specimens were then evaluated for microbial adhesion at each incubation time point (2, 4, 6 and 8 h). For the remaining analyses, after 1 h and 30 min of incubation under agitation in a bacteriological incubator, the specimens were washed twice with 2 mL of PBS and then received 1.5 mL of sterile culture medium in each well. The plates were incubated under agitation for 24 h in a bacteriological incubator. After this period, 1 mL of the culture medium was removed and replaced with 1 mL of fresh sterile medium in each well. Following an additional 24 h of incubation under agitation, the specimens were evaluated for microbial load (CFU/mL), biovolume (laser scanning confocal microscopy) and biofilm morphology and surface characteristics (scanning electron microscopy).

### 4.5. Microbial Load (CFU)

After 48 h of incubation [41], the specimens were rinsed, transferred to test tubes containing 10 mL of PBS, and then sonicated for 20 min in an ultrasonic bath with a 200 W power and a 40 KHz frequency to detach the biofilm. The test tubes were further agitated using a mechanical shaker, and serial dilutions ranging from 10^−^^1^ to 10^−^^4^ were prepared to make up each sample. Aliquots were plated on Sabouraud Dextrose Agar (for *C. albicans*), Rogosa Selective Lactobacillus (SL) Agar (for *L. casei*) and Mitis Salivarius Agar Base (for *S. mutans*) and incubated at 37 °C for 48 h in a microbiological incubator. Plates with culture media for *S. mutans* and *L. casei* were incubated under microaerophilic conditions. Colony counts were performed for each sample and the CFU/mL values were calculated considering dilution factors and inoculum volume in mL. Results were expressed as Log (CFU + 1).

### 4.6. Biovolume

After 48 h of incubation, specimens were rinsed with PBS solution and stained using Filmtracer™ LIVE/DEAD™ Biofilm Viability Kit (Molecular Probes, Inc., Eugene, OR, USA), following the manufacturer’s instructions. Fluorophores were excited at wavelengths of 488 nm and 540 nm, and images were obtained in the emission ranges of 550–770 nm and 610–630 nm using a magnification of 630×. Since biofilm formation is expected to present non-uniform thickness across the surface, five randomly selected regions per specimen were evaluated, and biofilm thickness was recorded for each and selected area. The Z-stack image series obtained using the Z-series scan function were used for three-dimensional reconstruction and subsequent assessment of biofilm biovolume.

### 4.7. Microbial Adhesion

After the incubation periods, the samples were rinsed with PBS and stained for 15 min using Filmtracer™ LIVE/DEAD™ Biofilm Viability Kit (Molecular Probes, Inc., Eugene, OR, USA), according to the manufacturer’s instructions. Specimens were rinsed twice with distilled water and analyzed under an inverted epifluorescence microscope using appropriate filters and a 630× magnification. ZEN 2.3 lite software (Carl Zeiss, Oberkochen, BW, Germany) was used to capture 20 random fields from each specimen surface. The images were subsequently analyzed using AxioVision software (AxioVision release 4.8.2-Carl Zeiss, Oberkochen, BW, Germany) to quantify the surface area covered by cells.

The results were expressed as a percentage (%) relative to the total image area [47]. The area covered by viable cells was determined by subtracting the area occupied by dead microorganisms (red) from the total biofilm area (green).

### 4.8. Scanning Electron Microscopy

Specimens were fixed in 2.5% buffered glutaraldehyde solution for 24 h, followed by dehydration in a graded ethanol solution series (30%, 50%, 70%, 95%, and 100%) for 30 min. Then, each specimen was subjected to three sequential 15 min immersions in Hexamethyldisilazane. Samples were subsequently sputter-coated with gold, and images were captured at 1000× and 5000× magnifications.

### 4.9. Statistical Analysis

Data were tested for normality using the Shapiro–Wilk test and for homogeneity of variances using Levene’s test. These assumptions were met for the microbial load (Log(CFU + 1)) of *Candida albicans*, *Lactobacillus casei*, dispersive energy, polar energy, and surface energy. Surface roughness (Sa) and wettability were analyzed using the Kruskal–Wallis test followed by Dunn’s post hoc test. For dispersive energy, polar energy, and surface energy, the groups were compared using one-way analysis of variance (ANOVA) followed by Tukey’s post hoc test. For the microbial load of *C. albicans* and *L. casei*, one-way ANOVA followed by Tukey’s post hoc test with Bonferroni adjustment was performed. For *Streptococcus mutans*, the Kruskal–Wallis test followed by Dunn’s post hoc test was applied. Microbial adhesion and biovolume were analyzed using the Wald test within the Generalized Estimating Equations (GEEs) model, followed by Bonferroni’s post hoc test. A significance level of 5% was adopted for all analyses. The software used was IBM Corp, released in 2012 (IBM SPSS Statistics for Windows, Version 21.0. Armonk, NY, USA: IBM Corp).

## 5. Conclusions

The results indicate that different surface finishing procedures for monolithic zirconia—polishing and glazing—significantly influence surface properties, including roughness, wettability, and surface free energy. However, no statistically significant differences were observed in biofilm formation and microbial adhesion.

## Figures and Tables

**Table 1:** Statistics of surface roughness (μm) and wettability (°), for the control, glazing, and polishing groups.

Group		Mean ± SD (Median)	95% CI (Range)
Roughness	Control	3.30 ± 1.2 (2.89)	2.50; 4.10 (2.45; 6.15)
	Glazing	13.24 ± 2.21 (13.69) *	11.67; 14.82 (8.92; 15.76)
	Polishing	2.28 ± 0.43 (2.39)	1.97; 2.59 (1.40; 2.97)
Wettability	Control	52.6 ± 11.3 (55.9)	44.6; 60.7 (34.7; 67.0)
	Glazing	15.0 ± 5.8 (12.2) *	10.9; 19.2 (9.5; 24.6)
	Polishing	46.3 ± 9.3 (43.1)	39.6; 53.0 (34.0; 60.7)

SD: standard deviation; CI: confidence interval. Range: minimum; maximum. * indicates a significant difference (*p* < 0.05), *n* = 10.

**Table 2:** Surface energy, dispersive energy and polar energy (mJ/m^2^) for the control, glazing, and polishing groups.

Group		Mean ± SD	95% CI (Range)
Surface Energy	Control	48.02 ± 4.58 ^A^	44.74; 51.29 (40.65; 55.74)
	Glazing	64.58 ± 2.05 ^B^	63.11; 66,05 (59.25; 66.39)
	Polishing	52.22 ± 3.89 ^C^	49.44; 55.00 (44.30; 56.95)
Dispersive energy	Control	26.79 ± 3.81 ^AB^	24.06; 29.52 (21.21; 32.00)
	Glazing	23.15 ± 3.18 ^A^	20.88; 25.43 (17.22; 28.13)
	Polishing	27.93 ± 2.87 ^B^	25.88; 29.99 (23.65; 32.82)
Polar energy	Control	21.23 ± 7.17 ^A^	16.09; 26.36 (12.84; 34.53)
	Glazing	41.43 ± 3.68 ^B^	38.79; 44.06 (37.30; 49.17)
	Polishing	24.29 ± 5.86 ^A^	20.09; 28.48 (14,39; 32.40)

SD: standard deviation; CI: confidence interval. Range: minimum; maximum. Letters ^A^, ^B^ and ^C^ indicate statistically significant differences (*p* < 0.05), *n* = 10.

**Table 3:** Descriptive statistics of total microorganisms in microbial adhesion (% of surface area) assays for the control, glazing, and polishing groups at 2, 4, 6, and 8 h.

Group						
	Control		Glazing		Polishing	
Time	Mean ± SD (Median)	95% CI (Range)	Mean ± SD (Median)	95% CI (Range)	Mean ± SD (Median)	95% CI (Range)
**2 h**	3.30 ± 4.99 (1.50)	0.96; 5.64 (0.31; 20.30)	2.38 ± 2.11 (1.67)	1.39; 3.37 (0.19; 8.55)	2.57 ± 4.95 (0.68)	0.25; 4.88 (0.06; 19.39)
**4 h**	6.69 ± 6.19 (4.33)	3.79; 9.58 (0.65; 23.73)	6.18 ± 6.66 (2.68)	3.06; 9.30 (0.51; 22.51)	4.05 ± 4.19 (2.11)	2.09; 6.00 (0.45; 14.95)
**6 h**	6.10 ± 3.36 (5.89)	4.52; 7.67 (0.83; 12.54)	3.92 ± 2.01 (3.91)	2.98; 4.86 (0.73; 7.02)	3.31 ± 2.76 (2.08)	2.02; 4.60 (0.38; 9.49)
**8 h**	8.94 ± 10.79 (3.01)	3.89; 13.99 (0.85; 36.47)	5.05 ± 3.77 (4.07)	3.29; 6.82 (0.92; 17.01)	3.24 ± 2.21 (2.76)	2.21; 4.27 (0.65; 7.59)

SD: standard deviation; CI: confidence interval for the mean, *n* = 2.

**Table 4:** Descriptive statistics of viable microorganisms in microbial adhesion (% of surface area) assays for the control, glazing and polishing groups at 2, 4, 6 and 8 h.

Group						
	Control		Glazing		Polishing	
Time	Mean ± SD (Median)	95% CI (Range)	Mean ± SD (Median)	95% CI (Range)	Mean ± SD (Median)	95% CI (Range)
**2 h**	3.16 ± 4.66 (1.50)	0.98; 5.34 (0.31; 18.80)	2.31 ± 2.08 (1.66)	1.33; 3.28 (0.19; 8.44)	2.41 ± 4.83 (0.68)	0.15; 4.67 (0.00; 19.04)
**4 h**	6.60 ± 6.11 (4.22)	3.75; 9.46 (0.63; 23.28)	6.12 ± 6.64 (2.64)	3.01; 9.23 (0.43; 22.45)	3.61 ± 4.06 (2.10)	1.71; 5.51 (0.43; 14.85)
**6 h**	5.93 ± 3.24 (5.77)	4.42; 7.45 (0.82; 12.49)	3.89 ± 2.01 (3.88)	2.95; 4.83 (0.71; 7.00)	3.23 ± 2.76 (2.02)	1.94; 4.52 (0.36; 9.43)
**8 h**	8.87 ± 10.77 (2.98)	3.82; 13.91 (0.83; 36.46)	5.00 ± 3.73 (4.02)	3.26; 6.75 (0.92; 16.71)	3.17 ± 2.23 (2.74)	2.12; 4.21 (0.62; 7.56)

SD: standard deviation; CI: confidence interval for the mean, *n* = 2.

**Table 5:** Descriptive statistics of dead microorganisms in microbial adhesion (% of surface area) assays for the control, glazing and polishing groups at 2, 4, 6 and 8 h.

Group						
	Control		Glazing		Polishing	
Time	Mean ± SD (Median)	95% CI (Range)	Mean ± SD (Median)	95% CI (Range)	Mean ± SD (Median)	95% CI (Range)
**2 h**	0.14 ± 0.36 (0.00)	0.00; 0.31 (0.00; 1.50)	0.08 ± 0.16 (0.01)	0.00; 0.15 (0.00; 0.61)	0.16 ± 0.29 (0.02)	0.02; 0.29 (0.00; 1.20)
**4 h**	0.08 ± 0.10 (0.05)	0.03; 0.13 (0.00; 0.45)	0.06 ± 0.06 (0.06)	0.03; 0.09 (0.00; 0.24)	0.43 ± 0.85 (0.08)	0.03; 0.83 (0.00; 3.34)
**6 h**	0.16 ± 0.34 (0.05)	0.00; 0.32 (0.00; 1.32)	0.03 ± 0.03 (0.02)	0.01; 0.04 (0.00; 0.10)	0.08 ± 0.09 (0.06)	0.04; 0.12 (0.00; 0.36)
**8 h**	0.07 ± 0.14 (0.02)	0.00; 0.14 (0.00; 0.64)	0.05 ± 0.09 (0.01)	0.01; 0.09 (0.00; 0.30)	0.07 ± 0.16 (0.03)	0.00; 0.15 (0.00; 0.72)

SD: standard deviation; CI: confidence interval for the mean, *n* = 2.

## Data Availability

The raw data supporting the conclusions of this article will be made available by the authors upon reasonable request.
